# Major Differences in the Diversity of Mycobiomes Associated with Wheat Processing and Domestic Environments: Significant Findings from High-Throughput Sequencing of Fungal Barcode ITS1

**DOI:** 10.3390/ijerph16132335

**Published:** 2019-07-02

**Authors:** Erika Yashiro, Dessislava Savova-Bianchi, Hélène Niculita-Hirzel

**Affiliations:** Center for Primary Care and Public Health (Unisanté), University of Lausanne, 1066 Lausanne, Switzerland

**Keywords:** mycobiome, bioaerosols, indoor, grain dust, rural, urban, farmers, harvesters, terminal elevator operators, wheat

## Abstract

Occupational exposure to grain dust is associated with both acute and chronic effects on the airways. However, the aetiology of these effects is not completely understood, mainly due to the complexity and variety of potentially causative agents to which workers are exposed during cereals process. In this study, we characterized the mycobiome during different steps of wheat processing—harvesting, grain unloading and straw handling—and compared it to mycobiomes of domestic environments—rural and urban. To do so, settled dust was collected at a six month interval for six weeks in the close proximity of 142 participants, 74 occupationally exposed to wheat dust—freshly harvested or stored—and 68 not occupationally exposed to it. Fungal community composition was determined in those samples by high-throughput sequencing of the primary fungal barcode marker internal transcribed spacer 1 (ITS1). The comparison of different mycobiomes revealed that fungal richness, as well as their composition, was much higher in the domestic environment than at the workplace. Furthermore, we found that the fungal community composition strongly differed between workplaces where workers handled freshly harvested wheat and those where they handled stored wheat. Indicator species for each exposed population were identified. Our results emphasize the complexity of exposure of grain workers and farmers and open new perspectives in the identification of the etiological factors responsible for the respiratory pathologies induced by wheat dust exposure.

## 1. Introduction

Occupational exposure to wheat dust has been shown to be associated with both acute and chronic effects on the airways of operators handling grain or straw [1,2]. However, the aetiology of these effects is not completely understood, mainly due to the complexity and variety of potentially causative agents within grain dust.

One way to clarify this issue is to focus on the populations exposed to one main type of crop dust. Wheat is the most intensively cultured cereal in western countries, and working populations handling wheat grain or straw are one such group that typically gets exposed to a single crop during a given period of time. The quantities of wheat generated require working populations with specialized task sets in the process of wheat harvesting and transformation. Harvest workers are specialized in grain or straw harvesting. Terminal elevator operators (TEOs) are specialized in grain unloading, cleaning, storage and loading. Finally, cattle raisers, by intensifying their activity, see an increase in their exposure to wheat straw as livestock litter. Although all of these populations are exposed to wheat dust, their level of exposure to this organic dust differs throughout the year. Harvesters and TEOs perform high exposure activities daily during wheat-harvesting season—from the beginning of July to the end of August in the Vaud region—when they unload grain and clean the installations, but the remainder of the year they have limited or no contact with transformed wheat [1]. In contrast, cattle raisers are exposed to wheat dust year round during the handling of stored wheat straw bales and the spreading of this straw as bedding for cows [1,3]. However this activity is more frequent from October to April than from April to October [2]. Interestingly, a significant difference has been observed in the clinical picture of the workers handling those two types of organic dust. The level of a recent exposure to field wheat dust was associated with an increased prevalence of five respiratory symptoms (cough, dyspnea, nasal congestion, scratchy throat, systemic symptoms) while that to stored wheat dust was only associated with an increased prevalence in coughing [1].

The dust components that are expected to change the most between the organic dusts delivered from freshly harvested or stored wheat, are the members of the fungal community associated with the fresh or stored plant material. Indeed, fresh wheat is known to be regularly infected by fungal pathogens, such as *Fusarium graminearum* that produces metabolites that are toxic for humans and animals [4,5], while stored wheat was described to be infected by other toxic and/or allergenic fungi, such as *Penicillium brevicompactum* and *Eurotium amstelodami*.

The aims of the present study were to determine the overall fungal community composition in the environment of all those different wheat-dust exposed populations, during the high and low exposure period, and to document the difference between the mycobiomes of wheat workers and those found in domestic environments—rural and urban.

## 2. Materials and Methods

### 2.1. Study Design

A longitudinal study was conducted on workers occupationally exposed to wheat dust (terminal elevator operators, harvesters, grain farmers and cattle raisers) and workers not occupationally exposed to it. The inclusion of the 142 participants to the overall protocol, the assessment of their exposure to bioaerosols, in particular to those associated to wheat dust, as well as the consequences of this exposure on respiratory symptoms and immune response, have been described in detail elsewhere [1,2]. Briefly, all participants were visited twice, at a six month interval, between August 2012 and June 2013. During the first visit, a detailed occupational history, including job title, workplace, start and stop dates, technological changes during their career, tasks undertaken in the previous six months with their duration and frequency, the collective and personal respiratory protective equipment used and plants handled was obtained by face-to-face questionnaire with each participant. At the second visit (V2), only the recent occupational exposure, including tasks undertaken within the last six months with their duration and frequency, plants handled and the collective and personal protective devices used was questioned. The workplace was systematically visited on V1 and V2 by an occupational hygiene specialist who estimated the exposure level of each participant to wheat dust during each wheat-related task in the previous six weeks based on a task-exposure matrix established in a previous study [2]. Doing high exposing activities—grain unloading, machines cleaning for harvesters and terminal elevator operators (TEO); intensive handling of stored wheat straw bales and spreading of this straw as bedding for cows for cattle raisers—has been associated with self-declared respiratory symptoms in those populations [3]. The period during which those activities occurred was called in the present work the “high exposure period”, although the one with low exposing activities (no associated symptoms) was called the “low exposure period”. The exposure of the control populations (rural and urban populations) was assigned as high or low exposure period when it was conducted during the same time periods. In order to identify which microorganisms might be responsible of this difference in the clinical picture, the settled dust was collected for six weeks with an electrostatic dust collector (EDC) [6] in the environment of each participant at the workplace where they handled wheat or at home when they did not. When the workers were exposed to wheat dust at different places, one EDC was installed at their closed proximity at each one of these places. Concerning the ethical code for our research, here is the information: The Human Research Ethics Committee from Vaud, Switzerland approved this study (Protocol 130/12). Written informed consent was obtained from all participants. All participants lived and worked in the Vaud region of Switzerland during the study.

### 2.2. Sample Collection and Treatment

The EDC was previously validated for fungal particles collection [7], fungal DNA extraction and amplification [8]. Thus, we followed already published protocols to wash the fungal particles from each EDC with a 0.1% Tween 80 solution [8], to mechanically disrupt them with a Tissue Lyser (Qiagen, Hilden, Germany) in the first buffer of the FastDNA Spin Kit for Soil (MP Biomedical, Zurich, Switzerland) and extract the total DNA [5].

The nuclear ribosomal internal transcribed spacer 1 (ITS1)—the formally adopted region as the primary fungal barcode marker [9]—was amplified using the forward primer ITS1F and the reverse primer ITS2 and sequenced on a GS FLX instrument with the FLX Titanium reagents at Microsynth (Balgach, Switzerland) as previously described [5]. Out of the 188 samples successfully amplified and sequenced, 113 were sampled during the high exposure period to wheat dust in the environment of 74 participants of and 74 samples during the low exposure period in the environment of 63 participants (Table 1).

### 2.3. Statistical Analysis

QIIME 1.8.0 [10]) was used for processing the raw demultiplexed pyrosequencing data. The fasta and quality files were quality controlled (split_libraries.py), the read orientation was adjusted (adjust_seq_orientation.py) and then chimeras were removed with the usearch settings (identify_chimeric_seqs.py and filter_fasta.py). The open reference OTU (operational taxonomic unit) picking was done (pick_open_reference_otus.py) using the UNITE dataset as reference (sh_refs_qiime_ver6_97_s_10.09.2014.fasta) and the prefiltering of reads set at 60% identity to known sequences. The taxonomy was assigned to the OTUs (assign_taxonomy.py) with the RDP classifier algorithm [11] and the UNITE dataset as the reference (sh_taxonomy_qiime_ver6_99_s_10.09.2014.txt). All of the samples were rarefied to 3000 reads per sample (single_rarefaction.py). The alpha diversity values were generated using alpha_diversity.py, while the bray_curtis_faith and binary_jaccard beta diversity matrices were generated using beta_diversity.py. These values were used for downstream statistical analysis.

Statistical analysis was implemented using the R framework (version 3.4.3) [12]. The significances of each environment during high and low exposure period on mycobiomes diversity were assessed using the ordination analysis and permutational multivariate analysis of variance (PERMANOVA) by the “Adonis” function in the R package “vegan” [13]. The indicator species analysis was done with the “indval” function in the R package “labdsv” [14]. *p*-value adjustment was done using the false discovery rate (FDR) method and the “p.adjust” function in R. During the summarization of the results, a strict cutoff of *p* < 0.05 was not obligatorily taken if there was a respectable divide between significant and non-significant adjusted *p*-value results. Given that with an OTU clustering resolution of 97%, fungal species of clinical importance sometimes became included into the same OTUs as non-pathogenic relatives and these OTUs sometimes were named by their non-pathogenic relative, representative sequences of OTUs belonging to the pathogen genera of interest were re-examined with blastn. A presence–absence table of these species was generated and used to build a heatmap as a function of worker occupation and sampling period. The differences in the relative abundance of a given species between groups were determined using the Kruskal–Wallis test.

## 3. Results

### 3.1. Differences in Fungal Community Composition Between Environments

**Figure 1:**
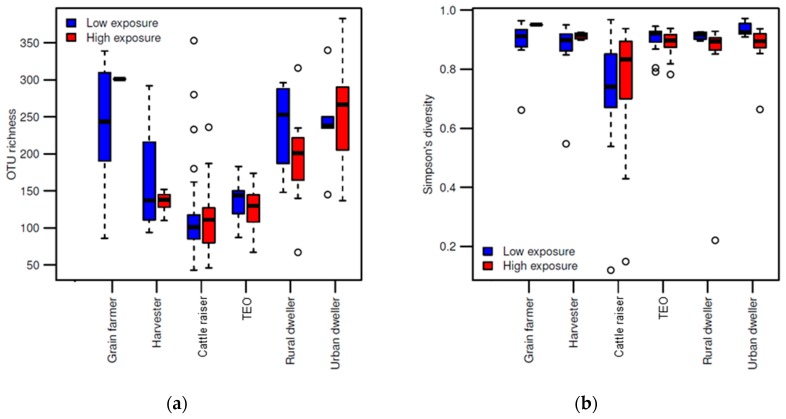
Fungal diversity among occupation groups according to the wheat dust exposure period: low or high. (**a**) Boxplot of OTU (operational taxonomic unit) richness among samples collected in the environment of each occupational group; (**b**) and of Simpson’s diversity among the same samples. The center line represents the median richness/diversity values, while the box represents the interquartile range and the whiskers the 1.5 times interquartile range.

After quality filtering and rarefying the samples to 3000 reads per sample, 4830 OTUs remained among 188 samples. The overall samples dataset was predominated by members belonging to the Ascomycota (59.62%) and Basidiomycota (31.89%), followed by unidentified fungi (8.29%) and Zygomycota (0.20%). The OTU richness within the fungal communities was generally associated with the respective professions of the workers, with cattle raisers, harvesters and terminal elevator operators (TEOs), exhibiting an exposure to a lower OTUs richness than grain farmers, rural and urban dwellers, independently of the sampling period (Figure 1a). The community diversity remained relatively uniform across the professions (Figure 1b). Only the cattle raisers displayed a higher variance in community diversity than individuals in other groups.

**Figure 2:**
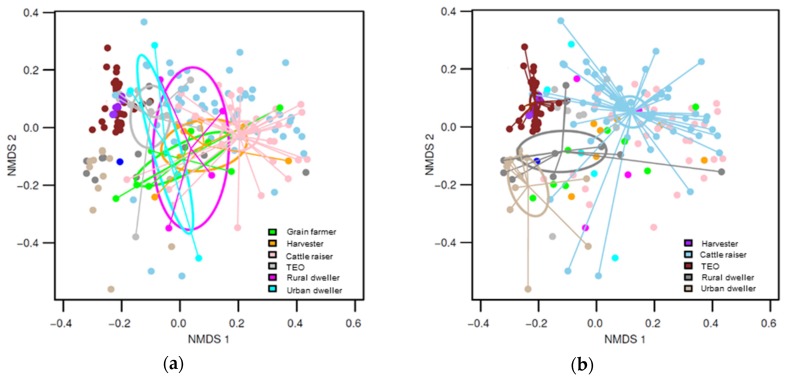
Nonmetric multidimensional scaling graph of fungal communities (stress = 0.1742681), with ordiellipses representing the 95% confidence intervals generated from the standard error, and the communities from each occupation group connected to the centroid by ordispider lines: (**a**) Highlights the communities sampled during the period of low exposure, whereas (**b**) highlights the communities sampled during the period of high exposure to wheat dust.

Although there were some innate differences in community composition among the groups (Table 2), during the low exposure period, the fungal community composition of the different groups remained relatively similar among each other and overlapped within the NMDS ordination space (Figure 2a). In contrast, during the high exposure period, the fungal community composition strongly differed among the worker populations (Figure 2b), as well as between the workplaces, notably where freshly harvested wheat was handled—harvesters and terminal elevator—and where stored wheat was handled—cattle raisers farms. A distinct pattern was also observed between the fungal communities sampled at the work place and those sampled at home in urban and rural dwellings at the same period (Figure 2b, Table 2, high exposure period vs. high exposure period). When the exposure to fungi was compared between the two sampling periods, a significant shift in the community composition was observed in the environment of terminal elevator operators, harvesters, cattle raisers and urban dwellers (Table 2, low exposure period vs. high exposure period).

### 3.2. Indicator Species and Fungi of Clinical Interest

**Figure 3:**
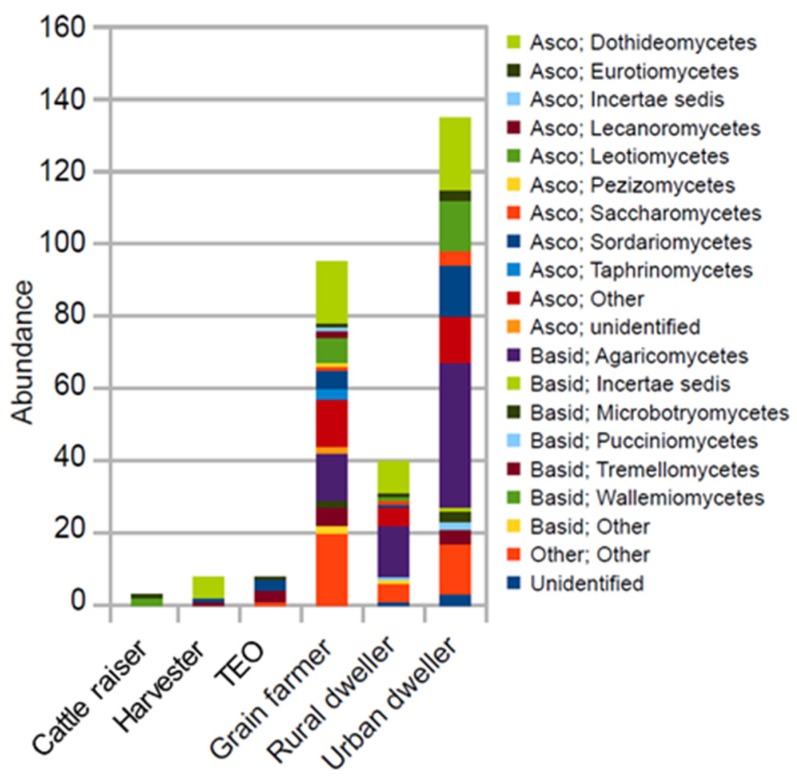
The mean of the relative abundances of each indicator OTU in the environment of each population of workers grouped at the fungal class level. The indicator OTUs were identified using Legendre and Legendre’s indicator species approach (R function “indval”). The abbreviations Basid and Asco represent the phyla Basidiomycota and Ascomycota, respectively

The indicator species analysis showed that OTUs belonging to six Basidiomycota, nine Ascomycota and other unidentified classes were enriched among specific groups of wheat workers and non-wheat-associated dwellers (Figure 3). Between three and 135 OTUs were identified for each group of individuals. Grain farmers, urban and rural dwellers were associated with more group-specific indicator OTUs than livestock, harvester and terminal operations workers. The fewer group-specific indicator OTUs among the latter groups of workers is also suggestive that they share many OTUs in common.

**Figure 4:**
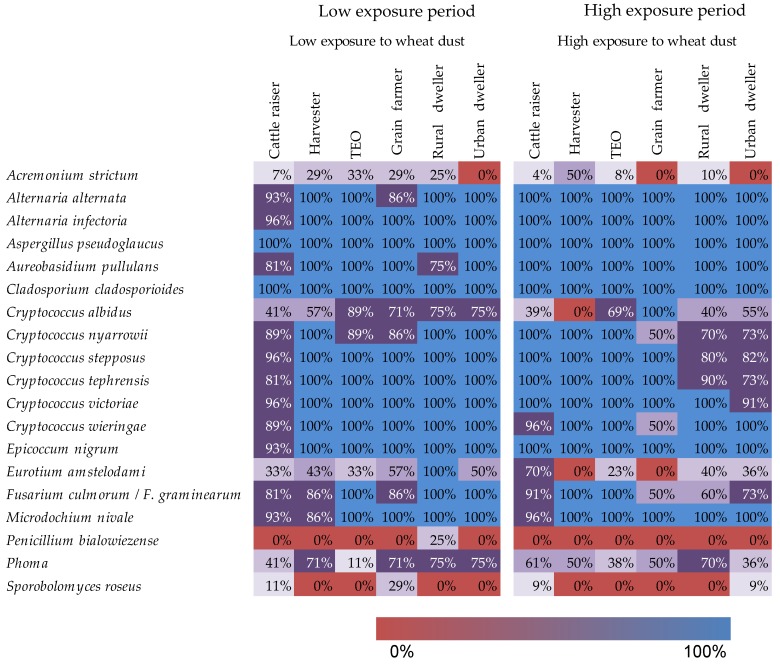
Heatmap illustrating the prevalence (i.e., presence–absence) of fungi of clinical importance, as a function of worker occupation and period of exposure to wheat dust. The colors and cell values indicate the proportion of samples in which the fungal species was detected.

The presence or absence of 19 fungi known for their allergenic, toxigenic or pathogenic effects was also specifically researched (Figure 4). 15 OTUs were prevalent among all of the groups during the two sampling periods, while only five—belonging to *Acremonium strictum, Cryptococcus albidus, Eurotium amstelodami, Penicillium bialowiezense* and *Sporobolomyces roseus*—were not detected in some of those environments, in particular in grain farmer and harvesters environment during the high exposure to wheat dust. The low number of samples collected for grain farmer and harvesters during the first sampling period might explain this finding. In contrast, *Eurotium amstelodami*, which was detected at a low incidence in the samples of most groups, was observed in 70% of cattle raisers environment during the high exposure period.

**Figure 5:**
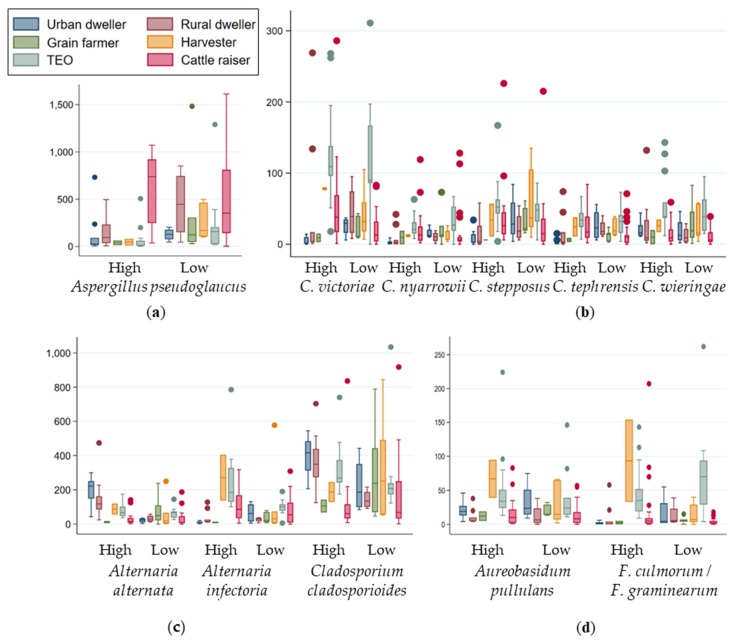
Examples of differences in exposure to fungal species of clinical importance among worker categories by exposure period, “High” indicates the high exposure period and “Low” the low exposure period: (**a**) Relative abundance of *Aspergillus pseudoglaucus* was much higher in cattle raisers during the period of high exposure; (**b**) relative abundance of five distinct *Cryptococcus* species; (**c**) relative abundance of *Alternaria infectoria* was higher at working places where wheat was handled, although that of *Alternaria alternata* and *Cladosporium cladosporioides* was higher in rural and urban dwellings; (**d**) relative abundance of *Aureobasidum pullulans* and *Fusarium culmorum/F. graminearum* was higher in harvesters’ environment during the high exposure period.

To determine whether or not the universally present fungi of clinical interest were equally abundant among the environment of the different groups of individuals, the relative abundances of these species were further examined. Generally, the relative abundances and variance in abundance were associated with respective worker groups and specifically associated with the level of exposure to wheat dust. Thus, during the period of high exposure to wheat dust, workers handling freshly harvested wheat—TEOs and harvesters—were significantly exposed to higher concentration of *Fusarium culmorum/F. graminearum*, *Microdochium nivale* and *Cryptococcus tephrensis (p* = 0.005, <0.001 and 0.003 respectively; Figure 5) than the other populations. The workers handling storage wheat—cattle raisers—were more exposed to *Aspergillus pseudoglaucus* and *Eurotium amstelodami* (*p* < 0.001 and 0.020; Figure 5a). The workers populations that did not handle wheat at the working place—rural and urban participants—were more exposed to *Alternaria alternata* and *Cladosporium cladosporioides* (*p* < 0.001; Figure 5c). In contrast, the wheat exposed working populations—TEOs, Harvesters and cattle raisers—were all more exposed to another *Alternaria* species: *Alternaria infectoria* (Figure 5c). Differences between the TEOs and harvesters exposure was also noticed concerning *Acremonium strictum* and *Cryptococcus wieringae* (Figure 5b), which were enriched in the harvesters’ environment (*p* < 0.001 and <0.001, respectively), and *Aureobasidum pullulans, Cryptococcus victoriae* and *Cryptococcus stepposus* in that of the TEOs (*p* = 0.003 and 0.015, respectively; Figure 5) During the low exposure period, only *Phoma* was found enriched in urban dwellings.

## 4. Discussion

This study showed the extreme difference between domestic and occupational exposure to fungi of cattle raisers, harvesters and terminal elevator operators (TEOs). At working places where wheat was handled, the fungal species richness was significantly lower than in a domestic environment—rural or urban. Moreover, the fungal communities were distinct between working and domestic environments, in particular during the high exposure period. During this period, even common species, such as *Alternaria alternata* and *Cladosporium cladosporioides*, were noticed at distinct relative abundances, these species being much more abundant in the domestic environment than at the working place. At the working place, other species of the same genera were more abundant—e.g., *Alternaria infectoria*. *Alternaria alternata* and *Cladosporium cladosporioides*—and which had been previously described to be highly frequent in outdoor and domestic environments during summer and autumn seasons [15], and *Alternaria infectoria* which had been associated with wheat [16]. However, this is the first time, to our knowledge, that these reported differences among *Alternaria* and *Cladosporium* species abundances have been clearly noticed within the same study.

Our study also highlights the major difference in workers’ exposure to fungi according to occupation. Cattle raisers were the only group to be exposed all throughout the year to species such as *Aspergillus pseudoglaucus* (= *Eurotium repens*) and *Eurotium amstelodami (= Aspergillus vitis)*, while harvesters and TEOs were seasonally exposed during the harvesting period to *Fusarium culmorum/F. graminearum*, *Microdochium nivale* and *Cryptococcus tephrensis*. Nevertheless, even harvesters and TEOs were found to be exposed to slightly different fungi such as *Cryptococcus* species, harvesters being more exposed to *Cryptococcus wieringae* while TEOs more to *Cryptococcus victoriae* and *Cryptococcus stepposus*. Most of those species were previously described in the environment of each respective working population, however they were not identified as indicator species of each one of those worker populations. Thus, *Fusarium culmorum/F. graminearum*, *Microdochium nivale* and *Cryptococcus wieringae* were previously described to be associated with freshly harvested wheat [5]. However, the exposure of the workers to those species was not characterized until now. The etiological relevance of at least one of those species—*Fusarium culmorum*/*F. graminearum*—is supported by in vitro experiments done on human respiratory cells [17]. Indeed, these *Fusarium* species were known to produce high quantities of mycotoxins—including deoxinivalenol, nivalenol and zearalenone—when they contaminated wheat. These concentrations were frequently high enough to be detected in aerosols [4], and to be cytotoxic for human respiratory cells [17]. *Cryptococcus* species—*Cryptococcus tephrensis, Cryptococcus victoriae* and *Cryptococcus wieringae*—were detected on Swedish cereal grain at harvest and a short time after storage [18,19]. The presence in larger quantities of some *Cryptococcus* species in grain workers’ environment than in cattle raisers’ one, can also make a difference in the clinical picture of those workers. Indeed, a distinct immune response against *Cryptococcus* of grain workers and cattle raisers has previously been described [1]. However, too few data are available to estimate the importance of exposure to different *Cryptococcus* species and the development of respiratory pathology. Another common contaminant of stored grains to which cattle raisers were specifically exposed was *Aspergillus pseudoglaucus*, a fungus known for its capacity to generate vast numbers of airborne spores and small mycelial fragments and a wide range of secondary metabolites—including cladosporin, echinulin and neoechinulin A and B—that are toxic for human respiratory cells [20]. Finally, *Eurotium amstelodami* has already been described to be highly frequent in dairy farmers’ environment [21]. However, its impact on farmers’ health is not supported yet by convincing epidemiological data.

These major differences in exposure to fungi among the general population, the population of workers handling field wheat—harvesters and TEOs—and the one handling stored wheat—cattle raisers—can explain the differences observed in the clinical picture among these populations. In this regard, in the healthy populations, the level of exposure to field wheat dust was associated with an increased prevalence of coughing, wheezing, dyspnea, nasal congestion, scratchy throat and systemic symptoms, while the level of exposure to stored wheat dust was associated with an increased prevalence in coughing [1].

The results of the present study must be interpreted in light of its strengths and weaknesses. First, our population size was relatively small, so we cannot exclude that some relationships between worker groups and exposure to fungi might have gone undetected because of the lack of power. Second, the study was designed to follow-up worker individual health with the level of exposure, between a season where they are highly exposed and another one where the exposure might be decreased, and to compare it to that of rural and urban groups. However, for controls these periods coincide with, respectively, summer and winter time. Consequently, the changes in mycobiome richness and composition observed for these groups correspond to a seasonal variation in their domestic exposure. Nevertheless, our study provides a comprehensive view on how different the mycobiomes present in the aerosols generated during the fresh or stored wheat handling are. Moreover, a main strength of this study is the choice of worker populations with clear exposure patterns.

## 5. Conclusions

The choice of worker populations with clear exposure patterns to wheat dust helped previously to define distinct acute and chronic health effects between grain workers and cattle raisers. In the present study, we showed that the fungal communities found in the wheat dust to which these populations were exposed, differed. Thus, we could distinguish fungal assemblages generated during the handling of fresh wheat from that of stored wheat material. Moreover differences were also noticed between periods of high and low exposure to wheat dust, as well as between wheat-related workers and those who were not directly involved with wheat crops in their daily lives. Furthermore, we were able to detect the prevalence of fungal pathogens of clinical importance that have typically been associated with fresh wheat as well as those pathogens associated with stored wheat on the respective worker populations. However, this information has to be collected in the environment of larger populations in order to identify the etiological factors responsible for the respiratory pathologies induced by wheat dust exposure. In the meantime, these results are sufficient to justify a limitation in the number and/or the intensity of the high exposure episodes to wheat dust for grain workers and cattle raisers.

## Figures and Tables

**Table 1:** Number of samples successfully amplified and sequences for each environment.

Worker Population	High Exposure			Low Exposure		
	*N* Samples	*N* Individuals	*N* Sites	*N* Samples	*N* Individuals	*N* Sites
Terminal elevator operator	25	18	7	11	11	8
Harvester	2	2	2	7	7	7
Grain farmer	2	2	2	8	8	8
Cattle raiser	56	24	24	39	28	28
Rural dweller	15	15	15	4	4	4
Urban dweller	13	13	13	5	5	5
Total	113	74	63	74	63	60

**Table 2:** Significant differences in the fungal diversity as a function of the period of exposure to wheat dust among worker groups. Results of the pairwise PERMANOVA analysis (Adonis).

PERMANOVA Pairs			F.Model	R^2^	*p*-Value	*p*-Adjusted
Low exposure period	vs.	High exposure period				
Cattle raiser	vs.	Cattle raiser	3.76	0.04	0.022	0.052
Harvester	vs.	Harvester	15.444	0.58	0.002	0.007
TEO ^1^	vs.	TEO	6.325	0.14	0.001	0.004
Urban dweller	vs.	Urban dweller	4.075	0.21	0.011	0.029
High exposure period	vs.	High exposure period				
Cattle raiser	vs.	Rural dweller	8.967	0.13	0.001	0.004
Harvester	vs.	Cattle raiser	16.741	0.23	0.001	0.004
Harvester	vs.	Rural dweller	8.659	0.35	0.001	0.004
TEO	vs.	Urban dweller	37.728	0.48	0.001	0.004
TEO	vs.	Cattle raiser	65.139	0.45	0.001	0.004
TEO	vs.	Rural dweller	19.793	0.33	0.001	0.004
TEO	vs.	Grain farmer ^2^	2.403	0.07	0.078	0.142
Urban dweller	vs.	Harvester	22.252	0.58	0.001	0.004
Urban dweller	vs.	Cattle raiser	17.217	0.22	0.001	0.004
Urban dweller	vs.	Rural dweller	2.71	0.11	0.034	0.073
Low exposure period	vs.	Low exposure period				
Cattle raiser	vs.	Urban dweller	7.328	0.17	0.002	0.007
Cattle raiser	vs.	Grain farmer	4.291	0.1	0.008	0.022
Cattle raiser	vs.	Rural dweller	2.477	0.06	0.069	0.129
Harvester	vs.	Cattle raiser	3.453	0.08	0.027	0.061
Harvester	vs.	Urban dweller	2.466	0.2	0.089	0.154
TEO	vs.	Harvester	5.469	0.26	0.006	0.017
TEO	vs.	Cattle raiser	25.821	0.38	0.001	0.004
TEO	vs.	Urban dweller	2.59	0.16	0.056	0.11
TEO	vs.	Grain farmer	5.699	0.25	0.004	0.012
TEO	vs.	Rural dweller	3.674	0.22	0.023	0.053

^1^ TEO: Terminal elevator operators; ^2^ grain farmer during high exposure period to wheat dust had only one sample.
